# Electrocardiographic abnormalities in COVID-19 patients visiting the emergency department: a multicenter retrospective study

**DOI:** 10.1186/s12873-021-00539-8

**Published:** 2021-11-19

**Authors:** Hugo De Carvalho, Lucas Leonard-Pons, Julien Segard, Nicolas Goffinet, François Javaudin, Arnaud Martinage, Guillaume Cattin, Severin Tiberghien, Dylan Therasse, Marc Trotignon, Fabien Arabucki, Simon Ribes, Quentin Le Bastard, Emmanuel Montassier

**Affiliations:** 1 Department of Emergency Medicine SAMU44, 44000 Nantes, France; 2 Department of Emergency Medicine, SAMU85, CHD La Roche Sur Yon, 85000 La Roche Sur Yon, France; 3 Department of Emergency Medicine, SAMU44, 44600 Saint Nazaire, France; 4 Cardiology, Centre Laroiseau, 56000 Vannes, France; 5 Department of Cardiology, CHD La Roche Sur Yon, 85000 La Roche Sur Yon, France

**Keywords:** Electrocardiogram, In-hospital mortality, Patterns, COVID-19

## Abstract

**Background:**

Severe acute respiratory syndrome coronavirus 2 (SARS-CoV-2) can be associated with myocardial injury. Identification of at-risk patients and mechanisms underlying cardiac involvement in COVID-19 remains unclear. During hospitalization for COVID-19, high troponin level has been found to be an independent variable associated with in-hospital mortality and a greater risk of complications. Electrocardiographic (ECG) abnormalities could be a useful tool to identify patients at risk of poor prognostic. The aim of our study was to assess if specific ECGs patterns could be related with in-hospital mortality in COVID-19 patients presenting to the ED in a European country.

**Methods:**

From February 1st to May 31st, 2020, we conducted a multicenter study in three hospitals in France. We included adult patients (≥ 18 years old) who visited the ED during the study period, with ECG performed at ED admission and diagnosed with COVID-19. Demographic, comorbidities, drug exposures, signs and symptoms presented, and outcome data were extracted from electronic medical records using a standardized data collection form. The relationship between ECG abnormalities and in-hospital mortality was assessed using univariate and multivariable logistic regression analyses.

**Results:**

An ECG was performed on 275 patients who presented to the ED. Most of the ECGs were in normal sinus rhythm (87%), and 26 (10%) patients had atrial fibrillation/flutter on ECG at ED admission. Repolarization abnormalities represented the most common findings reported in the population (40%), with negative T waves representing 21% of all abnormalities. We found that abnormal axis (adjusted odds ratio: 3.9 [95% CI, 1.1–11.5], *p* = 0.02), and left bundle branch block (adjusted odds ratio: 7.1 [95% CI, 1.9–25.1], *p* = 0.002) were significantly associated with in-hospital mortality.

**Conclusions:**

ECG performed at ED admission may be useful to predict death in COVID-19 patients. Our data suggest that the presence of abnormal axis and left bundle branch block on ECG indicated a higher risk of in-hospital mortality in COVID-19 patients who presented to the ED. We also confirmed that ST segment elevation was rare in COVID-19 patients.

**Supplementary Information:**

The online version contains supplementary material available at 10.1186/s12873-021-00539-8.

## Background

Severe acute respiratory syndrome coronavirus 2 (SARS-CoV-2) can be associated with myocardial injury, which have been described in various case reports, from acute myocarditis to pseudo acute myocardial infarction [1–4]. Emergency departments (EDs) worldwide have been at the epicenter of COVID-19 pandemic [5]. Early identification of cardiac involvement in COVID-19 in patients presenting to the Emergency Department (ED) is crucial. Electrocardiogram (ECG), widely performed in the ED and costless, could be a useful tool. Bertini et al. analysed COVID-19 patients who died or were treated with invasive mechanical ventilation, and found that electrocardiogram (ECG) recorded at hospital admission was abnormal in 93% of the patients, with signs of acute right ventricular pressure overload (RVPO) in 30% of the patients [6]. However, they did not compare these ECG findings to those from patients with mild to moderate forms of COVID-19. Mccullough and al. performed a retrospective cohort study in patients with COVID-19 who had an ECG at or near hospital admission in a large New York City teaching hospital. Using a multivariable logistic regression model that included age, ECG, and clinical characteristics, they found that the presence of one or more atrial premature contractions, a right bundle branch block or intraventricular block, ischemic T-wave inversion and nonspecific repolarization increased the odds of death [7]. However, these findings from a population with high incidence of cardiovascular conditions may be of limited external validity. Thus, the aim of our study was to assess if specific ECGs patterns could be related to in-hospital mortality in COVID-19 patients presenting to the ED in a European country.

## Methods

### Study design and participants

From February 1st to May 31st, 2020, we conducted a multicenter study in three hospitals in France: Nantes University Hospital, La Roche sur Yon Hospital and Saint Nazaire Hospital. We included adult patients (≥ 18 years old) who presented to the ED during the study period, with an ECG performed at ED admission, and diagnosed with COVID-19. COVID-19 diagnosis was confirmed by a positive reverse transcription-PCR (RT-PCR) targeting different genes of SARS-CoV-2 on nasopharyngeal swab [8]. All of the SARS-CoV2 PCR swabs were performed in ED.

### Data collection

Patients’ demographic details (age, gender), historical diagnoses and comorbidities (diabetes, coronary artery disease, history of cardiac heart failure, diabetes, arrhythmia, tobacco use, chronic obstructive pulmonary disease, chronic kidney disease (eGFR < 60 mL/min/m2), stroke, hypertension), current medication list (chronic oral anticoagulation, chronic antiplatelet therapy, anti-arrhythmia agents), signs and symptoms presented (blood pressure, heart rate, respiratory rate, oxygen flow rate, oxygen saturation, and chest pain), and outcome data were extracted from electronic medical records using a standardized data collection form. We used International Statistical Classification of Diseases and Related Health Problems, Tenth Revision, codes to define comorbidities in the population. Troponin T was assessed using Elecsys, Roche©. Two physicians (HDC and EM) checked the data extracted from said patients. Prior ECGs were rarely available on emergency room admission, which precluded comparison with ECGs performed on emergency room admission.

### Outcomes

The primary outcome was in-hospital mortality. We ascertained death based on review of discharge summaries and death notes in the electronic medical records.

### ECGs interpretation

Available ECGs were interpreted by two independent emergency physicians (Nantes University Hospital, Saint Nazaire Hospital) or by one emergency physician and one cardiologist (La Roche sur Yon Hospital) using a standardized data collection form [7]. When interpretation was discordant between the two physicians, another interpretation was performed by another independent cardiologist (DT). No formal testing of between- or within-reader variability of interpretation was performed for this study. Data extracted from each ECG included rhythm categorized as normal sinus rhythm or atrial fibrillation/flutter, atrioventricular block, axis deviation, intraventricular conduction block (IVB) (QRS duration of > 110 ms), right bundle branch block (RBBB), left bundle branch block (LBBB), left or right ventricular hypertrophy, ST segment or T-wave changes (localized ST elevation or depression, localized T-wave inversion, or other nonspecific repolarization abnormalities) and presence of U wave.

### Statistical analysis

Categorical variables are shown as frequency rates and percentages, and continuous variables as mean (standard deviation, SD). Relationship between ECG abnormalities and in-hospital mortality was assessed using univariate and multivariable logistic regression analyses, as described in additional file 1. Variables with a *p*-value under 0.10 were included for the multivariable model, and a backward regression was performed. Odds Ratio (OR) are expressed with 95% confidence interval. A two-sided α of less than 0.05 was considered statistically significant. Statistical analyses were done using the R software (version 3.6.0).

### Ethics

The study was approved by the Ethics Committee named Groupe Nantais d’Ethique dans le Domaine de la Santé, which waived the informed consent. Due to its retrospective nature on de-identified data, an informed consent was waived (GNEDS 28-05-2020). In France, the study is excluded from the legal requirements applicable to research involving humans within the provisions of the French Public Health Code. The sponsor of the study is CHU de Nantes (Nantes University Hospital), Delegation for Clinical Research and Innovation.

## Results

### Patient characteristics

A total number of 472 patients with COVID-19 were screened. Upon admission at ED, an ECG was performed on 275 patients who were further included in the analysis. Patients’ characteristics are shown in Table 1. The mean age was 70 ± 16 years old, of which 43% were women. Fifty-nine (21%) patients had chronic kidney disease, and 35 (13%) had diabetes mellitus. History of cardiovascular disease was common among the population: 42% had hypertension, 15% had congestive heart failure, and 14% had coronaropathy. We also found that 13% of the patients presented to the ED with chest pain, and 2% reported palpitations. Troponin was measured in 171 (62%) patients, with a mean level of 33 ± 59 ng/L. A Troponin level over 52 ng/L was found in 23 (8.4%) patients of whom 13 with a previous history of cardiac pathology.

**Table 1:** Characteristic of the studied population

Demographic characteristics	Survived (*n* = 238)	Died (*n* = 37)	Overall (*n* = 275)
Age, years (mean, SD)	68 ± 16	79 ± 10	70 ± 16
Male, n (%)	130 (55%)	27 (73%)	157 (56%)
Diabetes, n (%)	31 (13%)	4 (11%)	35 (13%)
Coronary artery disease, n (%)	33 (14%)	5 (14%)	38 (14%)
Heart failure, n (%)	37 (16%)	5 (14%)	42 (15%)
Atrial flutter and atrial fibrillation, n (%)	28 (12%)	11 (30%)	39 (14%)
Active smoker, n (%)	13 (5%)	2 (5%)	15 (5%)
Conduction disorders, n (%)	28 (12%)	4 (11%)	32 (12%)
Chronic kidney disease (eGFR < 60 mL/min/m2), n (%)	52 (22%)	7 (19%)	59 (21%)
Chronic Obstructive Pulmonary Disease, n (%)	20 (8%)	1 (3%)	21 (8%)
Hypertension, n (%)	98 (42%)	18 (49%)	116 (42%)
Stroke, n(%)	21 (9%)	6 (16%)	27 (10%)
Chronic oral anticoagulation, n(%)	39 (16%)	9 (24%)	48 (17%)
Chronic Antiplatelet therapy, n(%)	55 (23%)	9 (24%)	64 (23%)
Anti-Arrhythmia Agents, n(%)	19 (8%)	6 (16%)	25 (9%)
Vital signs, mean ± SD			
Systolic blood pressure, (mmHg)	135 ± 22	131 ± 26	134 ± 23
Diastolic blood pressure, (mmHg)	76 ± 14	73 ± 12	75 ± 14
Heart rate, (/min)	86 ± 16	84 ± 18	85 ± 16
Respiratory rate, (/min)	24 ± 6	27 ± 7	24 ± 6
Oxygen flow rate, (l/min)	1 ± 3	4 ± 5	1.5 ± 6
Oxygen saturation (%)	96 ± 3	96 ± 3	95 ± 3
Heart related symptoms, n (%)			
Chest pain	35 (15%)	0 (0%)	35 (13%)
Palpitations	6 (3%)	0 (0%)	6 (2%)
Outcome, n(%)			
In-hospital mortality	0 (0%)	37 (100%)	37 (14%)

Overall, eighty-seven (32%) patients received oxygen. In-hospital mortality was 14% (n = 37).

### Electrocardiographic findings

All 275 ECGs were interpreted by two emergency physicians or by an emergency physician and a cardiologist. A discordant interpretation between the two physicians was found in 41 (14.9%) ECGs. ECG findings are shown in Table 2. Baseline electrocardiographic characteristics included a mean heart rate of 85 ± 16 bpm, with a mean PR interval of 160 ± 40 ms and a mean QRS interval of 98 ± 29 ms. Most of the ECGs were in normal sinus rhythm (87%), and 26 (10%) patients had atrial fibrillation/flutter on ECG at ED admission. Abnormal axis was rare (*n* = 16, 6%), with 5% having left axis deviation and 1% a right axis deviation. Abnormal intraventricular conduction was found in 16% of the patients, with RBBB in 5% and LBBB in 4%. Repolarization abnormalities represented the most common findings reported in the population (40%), with negative T waves representing 21% of all abnormalities. Importantly, ST segment elevation was rare (*n* = 6%). When comparing patients with repolarization abnormalities to patients without, troponin levels were not significantly different (39 vs 31 ng/L, *p* = 0.45).

**Table 2:** ECG findings in the study population

	Survived (***n*** = 238)	Died (***n*** = 37)	Overall (***n*** = 275)
Sinus Rhythm, n (%)	211 (89%)	28 (12%)	239 (87%)
Abnormal axis, n (%)	11 (4%)	5 (14%)	16 (6%)
Heart rate, mean ± eSD	86 ± 16	84 ± 18	85 ± 16
Atrial fibrillation, n (%)	30 (13%)	5 (14%)	26 (10%)
Left atrial enlargement, n (%)	2 (1%)	1 (3%)	3 (1%)
Left ventricular hypertrophy, n (%)	3 (1%)	0	3 (1%)
Pacemaker rhythm, n (%)	3 (1%)	3 (8%)	6 (2%)
PR interval, mean ± SD	152 ± 46	181 ± 66	160 ± 40
QRS interval, mean ± SD	95 ± 27	106 ± 33	98 e± 29
Pathological Q wave, n (%)	29 (12%)	4 (11%)	33 (12%)
Any repolarization abnormality, n (%)	64 (27%)	8 (22%)	72 (26%)
Giant T wave, n (%)	14 (6%)	0 (0%)	14 (5%)
Pathological negative T wave, n (%)	52 (22%)	7 (19%)	59 (21%)
ST segment depression, n (%)	15 (6%)	1 (3%)	16 (6%)
ST segment elevation, n (%)	6 (3%)	0 (0%)	6 (2%)
First degree atrioventricular block, n (%)	14 (6%)	0 (0%)	14 (5%)
Left anterior hemiblock, n (%)	8 (3%)	3 (8%)	11 (4%)
Left Ventricular Hypertrophy, n (%)	3 (1%)	0 (0%)	3 (1%)
Delta wave, n (%)	2 (1%)	0 (0%)	2 (0.7%)
IVB, n (%)	32 (13%)	11 (30%)	43 (16%)
RBBB, n (%)	11 (5%)	2 (5%)	13 (5%)
LBBB, n (%)	6 (3%)	5 (14%)	11 (4%)
Ventricular extrasystoles, n (%)	11 (5%)	0 (0%)	11 (4%)
U waves, n (%)	14 (6%)	1 (3%)	15 (6%)
Poor R wave progression, n (%)	8 (3%)	0 (0%)	8 (3%)

*ECG* Electrocardiogram, *IVB* Intraventricular conduction block, *LBBB* Left bundle branch block, *RBBB* Right bundle branch block

### Relationship between electrocardiographic findings and outcomes

Univariate and multivariate logistic regression analyses were then performed (Table 3). Variables with a *p*-value under 0.10 in the univariate analysis were included for the multivariable model (sinus rhythm, Abnormal axis, IVB, LBBB), and a backward regression was performed, as detailed in the Additional file 1. We found that abnormal axis (adjusted odds ratio: 3.9 [95% CI, 1.1–11.5], *p* = 0.02), and LBBB (adjusted odds ratio: 7.1 [95% CI, 1.9–25.1], *p* = 0.002) were significantly associated with in-hospital mortality (Final model: Akaike Information Criterion: 206.5; Bayesian Information Criterion: 221.0; LROC: 0.64).

**Table 3:** Univariate and Multivariate logistic regression analysis of predictors of in-hospital mortality in hospitalized patients

	Survived (***n*** = 238)	Died (***n*** = 37)	Unajusted OR for in-hospital mortality, OR (95% CI); ***P*** value	Adjusted OR for in-hospital mortality, OR (95% CI); ***P*** value
sinus rhythm, n (%)	211 (89%)	28 (76%)	0.9 (0.8–1.0); 0.07	0.4 (0.2–1.2); 0.08
Abnormal axis, n (%)	11 (4%)	5 (14%)	1.21 (1.02–1.44); 0.03	3.9 (1.1–11.5); 0.02
Right atrial enlargement, n (%)	2 (1%)	1 (3%)	1.23 (0.83–1.80); 0.3	
Left atrial enlargement, n (%)	2 (1%)	1 (3%)	1.23 (0.83–1.80); 0.3	
Left ventricular hypertrophy, n (%)	3 (1%)	0	0.87 (0.64–1.18); 0.38	
Left anterior hemiblock, n (%)	8 (3%)	3 (8%)	1.13 (0.93–1.38); 0.22	
IVB, n (%)	32 (13%)	11 (30%)	1.16 (1.04–1.29); 0.008	
RBBB, n (%)	11 (5%)	2 (5%)	1.02 (0.85–1.24); 0.81	
LBBB, n (%)	6 (3%)	5 (14%)	1.4 (1.14–1.71); 0.001	7.1 (1.9–25.1); 0.002
Pathological Q waves, n (%)	29 (12%)	4 (11%)	0.99 (0.87–1.12); 0.85	
ST segment changes, n (%)	21 (9%)	1 (3%)	0.91 (0.79–1.05); 0.19	
Pathological negative T waves, n (%)	52 (22%)	7 (19%)	0.98 (0.89–1.08); 0.75	
Giant T wave, n (%)	14 (6%)	0	0.87 (0.73–1.04); 0.14	

*ECG* Electrocardiogram, *IVB* Intraventricular conduction block, *LBBB* Left bundle branch block, *RBBB* Right bundle branch block

## Discussion

Our multicenter study of the ECGs performed at ED admission in 275 COVID-19 patients found that repolarization abnormities were frequent, whereas ST segment elevation was rare, as previously reported [7]. In our cohort, the patients were older than in another Asian cohort [9], and our patients had lower prevalence rates of cardiovascular comorbidities than the North American cohorts [8, 10]. However, the baseline characteristics of COVID-19 patients reported here were consistent with those observed in French EDs during the first wave of COVID-19 pandemic [11]. Compared to a cohort of patients with community-acquired pneumonia (CAP), our population had similar age but with less cardiovascular risk factors [12]. Nonetheless, the comparison should be made with caution, regarding the impact of cardiovascular comorbidities in both COVID-19 and CAP.

In our multivariable logistic regression model, the presence of abnormal axis and LBBB were associated with in-hospital mortality. Finding early signs of cardiac impairment is extremely valuable in prioritizing ED patients, and our results suggest that ECG, widely performed in the ED and at no cost, is a useful tool in the ED assessment of COVID-19 patients. Mccullough and al. previously reported that ischemic T-wave inversion was associated with an increased risk of death in patients with COVID-19 who had a ECG at or near hospital admission [7]. Moreover, Lombardi et al. reported that elevated troponin was an independent variable associated with in-hospital mortality and a greater risk of complications during hospitalization for COVID-19 [13]. Myocardial involvement in COVID-19 is supported by pathological finding of interstitial inflammatory mononuclear cells in heart tissue during autopsy [14]. But the specific involvement of SARS-CoV2 is unclear in the underlying cardiac pathogenicity. Importantly, similar patterns have been found in CAP, where cardiovascular disease (CVD) events have been reported to be frequent [15–17] and ECG changes are often seen, with frequent ST segment or T-wave abnormalities [18]. Hypoxia, hypotension or decreased cardiac output could be one of the non-specific explanotary factors. Animal models have previously suggested that bacterial infections may lead to apoptosis of cardiomyocytes through direct and indirect toxicity of *S. pneumoniae* [19]. Violi et al. suggested that Nox2 related oxidative stress could explain myocardial damage [20]. Complex interactions through direct cardiomyocyte invasion and indirect inflammatory-mediated damage could explain cardiac involvement in COVID-19 [21]. Myocardial injuries have been described in various case reports, from acute myocarditis to pseudo acute myocardial infarction [1–4, 22], and several studies highlight an association between cardiac injuries and mortality [23–26]. Other studies suggested that abnormal heart rhythms are common [7, 13, 27]. But relationship between these findings and COVID-19 infection should be made with caution, as in an unselected population of healthy adults, inverted T waves in the anterior and lateral leads have been shown to be associated with long-term risk of mortality from CVD event [28]. A prospective cohort of patients with COVID-19 infection and ECG abnormalites should assess long-term cardiovascular consequences of COVID-19.

Our study has some limitations. First, because of its retrospective design, data were missing in both biological data and comorbidities, especially regarding troponin level which was available in only 171 patients. As symptoms onset was also unknown, this prevented us from doing any survival curve analysis. Since ECGs were not performed in every patient, it is possible that more severe patients had ECG analyses, therefore inducing a selection bias explaining the high mortality rate in our patients. Second, when the ECG was abnormal, it was not possible to determine if those abnormalities were related to the COVID-19 infection or to a previous unknown cardiopathy. Third, the ECG were interpreted by two emergency physicians or an emergency physician and a cardiologist, it is possible that some subtle ECG findings have been missed. However, previous studies suggested that most ED misinterpretations were determined unlikely to have clinical significance [29, 30]. Moreover, centralized ECG reading by cardiologists is not feasible in most hospitals. Fourth, echocardiography or cardiovascular magnetic resonance were not performed in all included patients for evaluation of heart involvement.

## Conclusions

In a multicenter study, we reported that ECG performed at ED admission may be useful to predict severity and death in COVID-19 patients. Our data suggest that the presence of abnormal axis and LBBB on ECG at ED admission indicated a higher risk of death. We also confirmed that ST segment elevation at ED presentation was rare in COVID-19 patients. A prospective study should be performed to evaluate the monitoring of ECG in COVID-19 prognosis, and if ECG abnormalities detected during COVID-19 infection is a risk factor for subsequent CVD event.

## Supplementary Information

**Additional file 1.** Details of the multivariate model performed

## Data Availability

The datasets used and/or analyzed during the current study are available from the corresponding author on reasonable request.

## Abbreviations

ECG: electrocardiogram
IVB: intraventricular conduction block
LBBB: left bundle branch block
RBBB: right bundle branch block
