# Supplementary material for: Electrocardiographic abnormalities in COVID-19 patients visiting the emergency department: a multicenter retrospective study
Source: BMC Emerg Med. 2021 Nov 19;21:141. doi: 10.1186/s12873-021-00539-8 (PMC8603337; doi:10.1186/s12873-021-00539-8)
Supplement: Supplementary file 1 — Additional file 1. Details of the multivariate model performed [file 12873_2021_539_MOESM1_ESM.docx]

**Additional file 1. Details of the multivariate model performed with R [1]**

############################################################

##########univariate analysis ########################################

############################################################

#Abnormal axis

reg <- glm(m$Died~ m$abnormal_axe)

summary(reg)

exp(cbind(coef(reg), confint(reg)))

#Right atrial enlargement

reg <- glm(m$Died~ m$HAD)

summary(reg)

exp(cbind(coef(reg), confint(reg)))

#Left atrial enlargement

reg <- glm(m$Died~ m$HAG)

summary(reg)

exp(cbind(coef(reg), confint(reg)))

#Left ventricular hypertrophy

sum(died$HVG)

reg <- glm(m$Died~ m$HVG)

summary(reg)

exp(cbind(coef(reg), confint(reg)))

#Left anterior hemiblock

reg <- glm(m$Died~ m$HBAG)

summary(reg)

exp(cbind(coef(reg), confint(reg)))

#IVB

reg <- glm(m$Died~ m$bloc_intraven)

summary(reg)

exp(cbind(coef(reg), confint(reg)))

#RBBB

reg <- glm(m$Died~ m$BBD)

summary(reg)

exp(cbind(coef(reg), confint(reg)))

#LBBB

reg <- glm(m$Died~ m$BBG)

summary(reg)

exp(cbind(coef(reg), confint(reg)))

#Pathological Q waves

reg <- glm(m$Died~ m$onde_Q)

summary(reg)

exp(cbind(coef(reg), confint(reg)))

#ST segment changes

reg <- glm(m$Died~ m$Anomalie_du_segment_ST_0.non_1._oui)

summary(reg)

exp(cbind(coef(reg), confint(reg)))

#Pathological negative T waves

reg <- glm(m$Died~ m$ondes_T_négatives)

summary(reg)

exp(cbind(coef(reg), confint(reg)))

#Giant T wave

reg <- glm(m$Died~ m$onde_T_amples_et_positives)

summary(reg)

exp(cbind(coef(reg), confint(reg)))

############################################################

########## multivariate ########################################

############################################################

# Variables with a p-value under 0.10 were included for the multivariable model (sinus rhythm, Abnormal axis, IVB, LBBB), and a backward regression was performed.

glm_full <- glm(Died ~ sinus_rhythm

LBBB +

IVB +

abnormal_axis, data=m, family=binomial(link = "logit") )

backwards <- step(glm_full, direction = "backward", trace = 1)

summary(backwards)

Start: AIC=207.43

Died ~ sinus_rhythm

LBBB +

IVB +

abnormal_axis

Df Deviance AIC

- IVB 1 198.55 206.55

- sinus_rhythm 1 199.40 207.40

<none> 197.43 207.43

- LBBB 1 201.00 209.00

- abnormal_axis 1 202.46 210.46

Step: AIC=206.55

Died ~ sinus_rhythm + LBBB + abnormal_axe

Df Deviance AIC

<none> 198.55 206.55

- sinus_rhythm 1 201.26 207.26

- abnormal_axis 1 203.27 209.27

- LBBB 1 206.40 212.40

exp(cbind(coef(backwards), confint(backwards)))

AIC(backwards)

BIC(backwards)

**Reference**

[1] R Core Team (2021). R: A language and environment for statistical computing. R Foundation for Statistical Computing, Vienna, Austria. URL https://www.R-project.org/
